# YLT192, a Novel, Orally Active Bioavailable Inhibitor of VEGFR2 Signaling with Potent Antiangiogenic Activity and Antitumor Efficacy in Preclinical Models

**DOI:** 10.1038/srep06031

**Published:** 2014-08-12

**Authors:** Yong Xia, Xuejiao Song, Deliang Li, Tinghong Ye, Youzhi Xu, Hongjun Lin, Nana Meng, Guobo Li, Senyi Deng, Shuang Zhang, Li Liu, Yongxia Zhu, Jun Zeng, Qian Lei, Youli Pan, Yuquan Wei, Yinglan Zhao, Luoting Yu

**Affiliations:** 1 State Key Laboratory of Biotherapy and Cancer Center, West China Hospital, West China Medical School, Sichuan University, Chengdu, 610041, China; 2 Department of Pathophysiology, School of Basic Medicine, AnHui Medical University, Hefei, 230032, China; 3 These authors contributed equally to this work.

## Abstract

Antagonizing vascular endothelial growth factor receptor 2 (VEGFR2) to block angiogenesis has been applied toward cancer therapy for its role in promoting cancer growth and metastasis. However, most these clinical anticancer drugs have unexpected side effects. Development of novel VEGFR2 inhibitors with less toxicity remains an urgent need. In this study, we describe a novel, well-tolerated, and orally active VEGFR2 inhibitor, YLT192, which inhibits tumor angiogenesis and growth. YLT192 significantly inhibited kinase activity of VEGFR2 and suppressed proliferation, migration, invasion, and tube formation of human umbilical vascular endothelial cells (HUVEC) *in vitro*. In addition, it inhibited VEGF-induced phosphorylation of VEGFR2 and its downstream signaling regulator in HUVEC. Zebrafish embryonic models and alginate-encapsulated tumor cell assays indicated YLT192 also inhibited angiogenesis *in vivo*. Moreover, YLT192 could directly inhibit proliferation and induce apoptosis of cancer cells *in vitro* and *in vivo*. Oral administration of YLT192 at a dose of 100 mg/kg/day could markedly inhibited human tumor xenograft growth without causing obvious toxicities. It decreased microvessel densities (MVD) in tumor sections. It also shows good safety profiles in the studies with mice and rats. Taken together, these preclinical evaluations suggest that YLT192 inhibits angiogenesis and may be a promising anticancer drug candidate.

Inducing angiogenesis is one of the hallmarks of cancer. New blood vessels infiltrate tumors, furnishing them with oxygen and nutrients, and provide a route for tumor metastasis^1^. Therefore, therapy based on blocking angiogenesis may be an effective strategy for inhibiting tumor growth and metastasis^2^.

Numerous growth factors and cytokines are involved in angiogenesis. Among all the known angiogenic molecules, vascular endothelial growth factor (VEGF) and its receptors (VEGFR) are pivotal stimuli of physiologic and pathologic angiogenesis^3^,^4^. VEGF binds to specific transmembrane receptors on endothelial cells (ECs) and lymphatic vessels and leads to phosphorylation of various downstream signal transduction proteins, including mitogen-activated protein kinase (MAPK), focal adhesion kinase (FAK), Src kinase, signal transducer and activator of transcription-3 (STAT3) and protein kinase B (AKT)^5^,^6^,^7^. There are five members in the VEGFR family and the main subtypes are VEGFR1–3, which are mainly located on the surface of ECs of normal tissues and only overexpressed during embryonic and tumor angiogenesis. VEGFR2 is the major effector for execution of VEGF-stimulated cell proliferation, vascular permeability, cell migration, and cell survival, leading to angiogenesis^4^. The production of VEGF is disproportionately elevated because of oncogene activation^8^, loss of tumor suppressor function^9^,^10^, or changes in oxygen or glucose status^11^,^12^. Therefore, VEGFR2 is strongly autophosphorylated in tumors by elevated VEGF expression.

With the exception of female reproduction tissues, angiogenesis does not occur in healthy adults. Compared with chemotherapy, which causes severe adverse effects and leads to drug resistance with long-term treatment, therapy aimed at inhibiting angiogenesis may be effective at preventing tumor progression and is well-tolerated^13^. Given that VEGFR2 plays a predominant role in promoting angiogenesis, it's the most important target in anti-angiogenesis therapy against cancer. A number of small molecule VEGFR2 inhibitors have been reported, including some Food and Drug Administration approved drugs such as sunitinib, sorafenib, and vandetanib^14^,^15^,^16^. Moreover, there are more small molecule inhibitors targeting VEGFR2 under clinical and preclinical evaluation, such as isoliquiritigenin^17^ and NBM-T-BMX-OS01^18^. However, adverse effects have been observed during clinical use or clinical trials, such as bleeding complications^19^, indicating that development of safer VEGFR2 inhibitors is still needed.

Our aim was to develop a novel small molecule VEGFR2 inhibitor that potently and specifically blocks the VEGF/VEGFR2 signaling cascade system with little or no toxicity. In the present study, we describe a novel synthetic compound, YLT192. In *in vitro* assays, YLT192 inhibited VEGF-induced VEGFR2 phosphorylation and activation of downstream signaling transduction mediators at relatively low concentrations. Based on its molecular mechanism, it significantly inhibited EC proliferation, migration, invasion, and tube formation. Cellular thermal shift assay also validated that YLT192 bind to VEGFR2. Moreover, it exhibited the ability to inhibit angiogenesis in the alginate-encapsulated tumor cell assay and zebrafish embryonic angiogenesis models. YLT192 directly inhibited cancer cell proliferation and migration, induced cell apoptosis, and blocked the oncogenic signaling pathways in tumor cells. Furthermore, this compound had excellent pharmacokinetic profiles that made it suitable for chronic once-daily oral administration *in vivo*. YLT192 dose-dependently inhibited the growth of tumor xenografts in athymic mice without causing obvious off-target toxicities in the host. Taken together, our data suggested that YLT192 could function as a novel, well-tolerated, and potent VEGFR2 inhibitor that suppresses tumor angiogenesis and growth.

## Results

### Kinase inhibition profile and molecular models of YLT192

**Figure 1:**
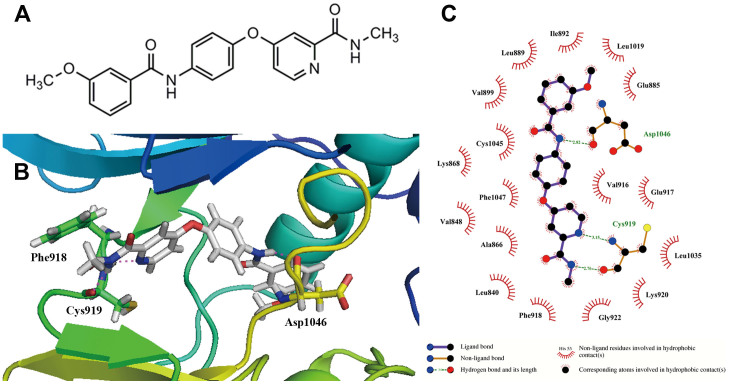
(A) Chemical structure of YLT192. (B) YLT192 is docked into the active site of VEGFR2, showing interactions between YLT92 and VEGFR2 in the 3-dimentional structure. (C) A 2-dimentional interaction map of YLT192 and VEGFR2.

YLT192 was designed and synthesized in the State Key Laboratory of Biotherapy, Sichuan University (Sichuan, China). Its structural formula and synthesis route was shown in Fig. 1A and Supplementary Figure S1, respectively. The kinase inhibitory activity of YLT192 was measured by the use of radiometric assays provided by Kinase Profile Service (Millipore, UK). The effects of YLT192 on kinase activity were detected using the scintillation proximity assay method at an enzymatic level. As shown in Table 1, YLT192 exhibited great inhibitory activity on VEGFR2 with an inhibitory rate of 93% at 1 μM. In addition, the inhibitory activity of YLT192 was examined against platelet-derived growth factor receptor (PDGFR)-related kinases because of their structural similarity to the VEGFR2. YLT192 showed a relatively low inhibitory rate of 0%, 5%, 7%, and 6% against Fms-like tyrosine kinase 3, PDGFRα, PDGFRβ, and cKit at 1 μM, respectively. Moreover, excellent selectivity for VEGFR2 was evident compared with a range of unrelated tyrosine and serine/threonine kinases, including fibroblast growth factor receptor 3, polo-like kinase 1, aurora-A, Axl, MET, protein kinase A, c-RAF etc. These results indicated that YLT192 was a potent VEGFR2 inhibitor.

We used molecular docking to analyze the binding mode of YLT192 with the inactive conformation of VEGFR2. A total of 20 docking poses were generated by GOLD program. According to the RMSD values, two docking pose clusters (cluster I and cluster II) were obtained. Cluster I contains 18 docking poses, while cluster II only has 2 docking poses. Therefore, we considered that the docking poses in cluster I might be the true binding modes of YLT192. Figure 1B and 1C depict the binding pose of YLT192 with the highest Goldscore value of 67.1791. The N-methyl picolinamide of YLT192 is observed to form strong hydrogen-bonding interactions with the Cys919 residue in the hinge region of VEGFR2. The nitrogen atom in the amide moiety of YLT192 forms another important hydrogen-bonding interaction with the Asp1046 residue in the DFG region of the VEGFR2. In addition, YLT192 forms hydrophobic interactions with residues Ile898, Leu889, Val899, Ile892, and Val899 in the allosteric pocket of VEGFR2.

### YLT192 inhibited human umbilical vascular endothelial cell (HUVEC) proliferation, migration, invasion, and tube formation

The inhibition effect of YLT192 on VEGF_165_-stimulated HUVEC growth was examined using MTT assay. The results showed that YLT192 inhibited HUVEC proliferation with an IC_50_ of 2.30 μM induced by VEGF_165_ (50 ng/mL), indicating YLT192's inhibitory effect on VEGFR2-dependent HUVECs proliferation. In addition, 5-ethynyl-2′-deoxyuridine (EdU) incorporation assays showed that YLT192 inhibited VEGF_165_-stimulated HUVEC proliferation (Supplementary Fig. S2).

**Figure 2:**
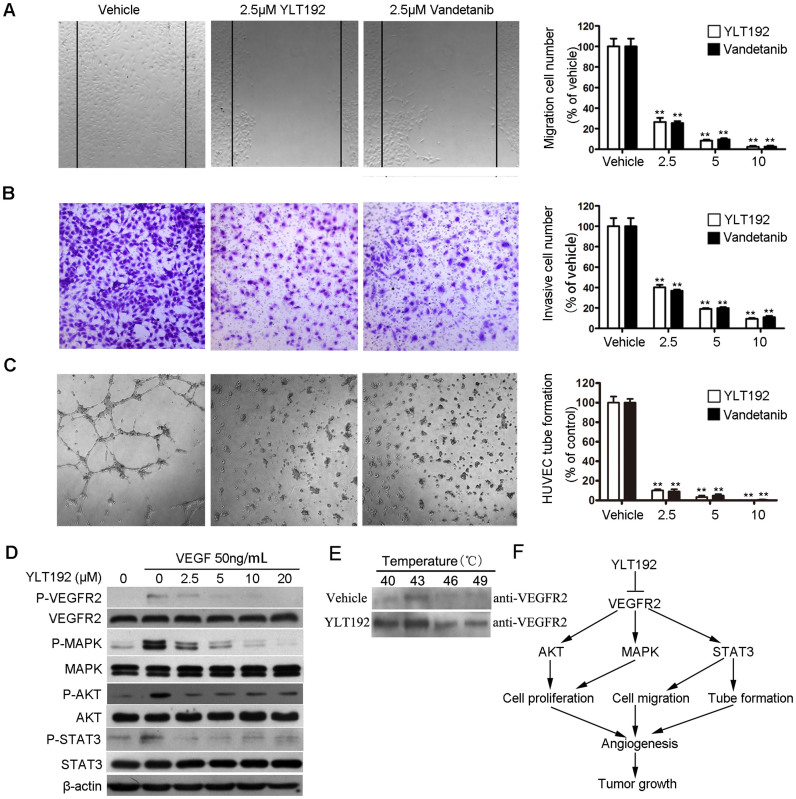
Effects of YLT192 on HUVECs proliferation, migration, invasion and tube formation. (A) YLT192 and Vandetanib inhibited HUVECs migration in wound healing assay. Cells were wounded by the pipette and then treated with various concentrations of compounds for 24 hours. Migrated cells were quantified by manual counting (50×). (B) YLT192 and Vandetanib inhibted HUVECs invasion in transwell assay. The bottom chambers of the transwells were filled with 600 μL EGM2 containing various growth factors while the top chambers were seeded with 4 × 10^4^ HUVECs in EBM2 and treated with different concentrations of compounds for 24 hours. Cells invaded through the membrane were stained and quantified (100×). (C) YLT192 and Vandetanib inhibited HUVECs tube formation. HUVECs were seeded on Matrigel layer and treated with different concentrations of compounds. HUVECs tube formation was assessed 6 hours later (50×). One representative experiment of three is shown. For (A), (B) and (C), data represent the mean ± SEM of three different experiments. Each experiment was performed in duplicate.*P < 0.05, **P < 0.01. (D) YLT192 inhibited VEGF-induced phosphorylation of VEGFR2 and its downstream signal regulator p44/42 MAPK, STAT3 and AKT in HUVECs. One representative Western blot of three is presented. Each has the expression of β-actin as internal control. The full size blots were shown in the Supplemental Figure S5 online. (E) Cellular thermal shift assay showing VEGFR2 target engagement by YLT192 in intact HUVECs. One representative Western blot of two is presented. The full size blots were shown in the Supplemental Figure S6 online. (F) Proposed model of YLT192 functions in inhibiting tumor angiogenesis and tumor growth through VEGFR2-mediated signaling pathways.

Cell migration is essential for ECs in angiogenesis and for cancer cells in tumor growth and metastasis^20^,^21^. We performed wound healing assays to investigate the effects of YLT192 on cell migration and observed 2.5 μM YLT192 strongly inhibited the migration of HUVEC (Fig. 2A). Cell invasion is essential for EC in angiogenesis, so we performed transwell assays to evaluate the ability of HUVEC to pass through the Matrigel and membrane barriers of the transwell in the presence of various concentrations of YLT192 or vehicle. As shown in Figure 2B, YLT192 significantly inhibited the invasion activities of HUVECs in a concentration-dependent manner.

To elucidate the possible mechanisms of angiogenesis inhibition, tube formation ability of ECs, which is a critical step in the process of angiogenesis, was assessed in HUVEC *in vitro*. As shown in Figure 2C, HUVEC plated on the surface of Matrigel formed capillary-like structures in the vehicle group within six hours. However, treatment with designed concentrations of YLT192 strongly inhibited the tube formation of HUVEC.

As shown in Figure 2, the results indicated that YLT192 had similar antiangiogenic effects in HUVEC migration, invasion, and tube formation in comparison with the clinically used VEGFR2 inhibitor Vandetanib.

### YLT192 inhibited VEGF-induced VEGFR2 phosphorylation in HUVEC

The phosphorylation of VEGFR2 and its downstream protein kinase stimulates angiogenesis. We investigated the effects of YLT192 on VEGFR2 signaling pathway in HUVEC. As shown in Figure 2D, YLT192 clearly reduced VEGF-stimulated phosphorylation of VEGFR2 and downstream p44/42 MAPK, AKT, and STAT3 in HUVEC in a concentration-dependent manner. In contrast, total levels of VEGFR2, p44/42 MAPK, AKT, and STAT3 were not affected by YLT192 treatment.

Then we investigate whether YLT192 bind to VEGFR2 in intact HUVECs. If VEGFR2 is bound by YLT192, it will be stabilized by the physical engagement^22^. In the cellular thermal shift assay, YLT192 efficiently stabilized VEGFR2, validating YLT192 is targeted to VEGFR2 in HUVECs (Fig. 2E).

### YLT192 inhibited angiogenesis *in vivo*

**Figure 3:**
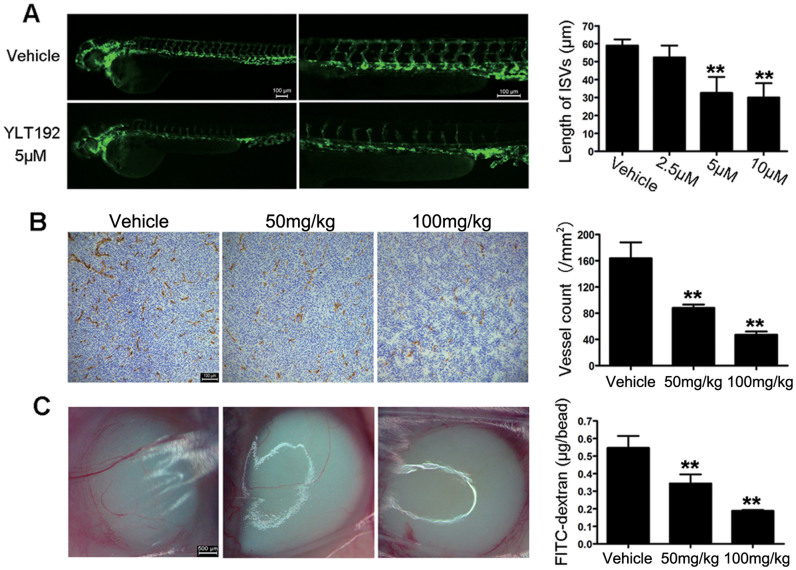
YLT192 inhibited angiogenesis *in vivo*. (A) Fluorescent images of 30 hpf zebrafish treated with vehicle or concentrations of YLT192. ISV growth was inhibited by YLT192. One representative experiment of three is shown. Data are means ± SEM (n = 10 per group), *P < 0.05, **P < 0.01. Scale bars represent 100 μm. (B) YLT192 significantly inhibited tumor microvessels in HCT116 tumor xenografts. Frozen sections of HCT116 tumors were tested by immunohistochemistry analysis with anti-CD31 antibody. Representative images of the tumor vasculature from vehicle and YLT192 treated mice were shown. The fields were averaged in each tumor, and the averages for each animal were used to give the final mean ± SD (n = 6 mice per group). *P < 0.05, **P < 0.01. Scale bars represent 100 μm. (C) YLT192 significantly inhibited angiogenesis in mice with alginate beads containing CT26 tumor cells. Alginate beads containing 5 × 10^4^ CT26 cells were implanted s.c. into the backs of mice. Mice were then orally treated with YLT192 or vehicle once daily. Beads were surgically removed 14 days later and FITC-dextran was quantified. FITC-dextran uptake of beads from YLT192 treated mice showed a significant decrease compared with vehicle treated group. Data are means ± SD (n = 6 mice per group), *P < 0.05, **P < 0.01. Scale bars represent 500 μm.

To test the anti-angiogenesis effects of YLT192 *in vivo*, we used a FLK-1 promoter enhanced green fluorescent protein (EGFP) transgenic zebrafish model. As shown in Figure 3A, 5 μM YLT192 considerably blocked the formation of intersegmental vessels compared with vehicle-treated embryos, whereas the dorsal aorta and major cranial vessels were not inhibited, indicating the antiangiogenesis effects of YLT192 *in vivo*.

Anti-CD31 immunohistochemical analysis of the tumor sections from HCT116 xenograft models revealed that YLT192 significantly reduced the microvessel density (MVD) after YLT192 treatment. As shown in Figure 3B, compared with the vehicle-treated group, MVD was reduced by 69% after treatment of 100 mg/kg YLT192 in HCT116 xenograft models. In addition, antiangiogenic effects were observed in CT26-induced alginate-encapsulated tumor cell assays. As shown in Figure 3C, new blood vessels in alginate beads were apparently inhibited in 100 mg/kg YLT192-treated mice compared with the vehicle-treated group. Moreover, the accumulation of fluorescein isothiocyanate (FITC)-dextran remarkably decreased after YLT192 treatment and 100 mg/kg YLT192 treatment reduced the accumulation of FITC-dextran by 66%. These data revealed that YLT192 inhibited tumor angiogenesis *in vivo*, which aided in suppressing tumor growth.

### YLT192 inhibited tumor cell growth *in vitro*

**Figure 4:**
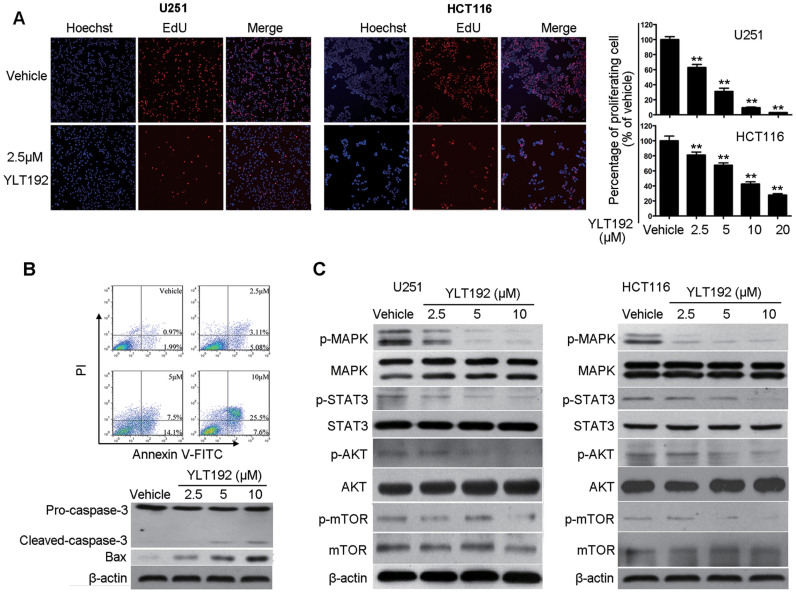
Inhibitory effects of YLT192 on tumor cells. (A) YLT192 inhibited U251 and HCT116 cells proliferation. The EdU incorporation assay was conducted on U251 and HCT116 cells after treated with YLT192 for 24 hours. The EdU-positive (red fluorescent staining) cells and Hoechst staining (blue fluorescent staining) cells represented proliferating and total cells in the fluorescent images, respectively (100×). Quantification was shown on the right panels. (B) YLT192 induced apoptosis of HCT116 cells. HCT116 cells were treated with YLT192 or vehicle for 48 hours followed by flow cytometric analysis of annexin V-FITC and PI staining. The proportion of early apoptotic cells (annexin V positive) and late apoptotic cells (annexin V and PI positive cells) were shown. Typical apoptosis-related proteins caspase 3 and Bax were detected by western blot. Each has the expression of β-actin as internal control. The full size blots were shown in the Supplemental Figure S6 online. For (A) and (B), one representative experiment of three is shown. Data are means ± SEM, *P < 0.05, **P < 0.01. (C) YLT192 inhibited oncogenic signaling pathways in tumor cells *in vitro*. U251 and HCT116 cells were treated with YLT192 or vehicle for 48 hours and the expression of protein in different signaling pathways were detected by western blot with specific antibodies. Blots are representative of three experiments. Each has the expression of β-actin as internal control. The full size blots were shown in the Supplemental Figure S7 online.

The proliferative inhibitory ability of YLT192 on tumor cells was investigated. Most IC_50_ against the tumor cells ranged from 6 to 20 μM, showing remarkable inhibition of tumor cell viability (Supplementary Table S1). Inhibition of cell proliferation is a possible approach for cancer treatment^23^. EdU incorporation assay was performed to investigate the anti-proliferative effects of YLT192 against U251 and HCT116 cells. As shown in Figure 4A, the percentage of proliferating U251 and HCT116 cells was decreased a lot after 24 h of treatment with YLT192, indicating that YLT192 could significantly inhibit the proliferation of U251 and HCT116 cells in a concentration-dependent manner.

Annexin V/propidium iodide staining was used to perform the cell apoptosis assay to examine the effects of YLT192 on tumor cell apoptosis. As shown in Figure 4B, treatment with YLT192 increased apoptosis in HCT116 cells in a concentration-dependent manner. Western blot analysis showed a reduction of pro-caspase-3 and an increase in levels of its pro-apoptotic cleaved forms after treatment with YLT192 in HCT116 cells. In addition, we observed that YLT192 increased the expression of pro-apoptotic Bax. These data indicated that YLT192 could induce apoptosis in HCT116 cells.

To gain further insight into the molecular mechanism of the inhibitory effects of YLT192 on tumor cells we evaluated the expression of p44/42 MAPK, STAT3, AKT, and mammalian target of rapamycin (mTOR) in U251 and HCT116 cells 48 h after treatment. As shown in Figure 4C, YLT192 dramatically decreased the expression of phosphorylated p44/42 MAPK, STAT3, AKT, and mTOR, whereas the total protein levels of each protein were not significantly changed.

### Pharmacokinetic properties of YLT192

Appropriate pharmacokinetic parameters are essential to determine the efficacy of anti-cancer drugs. We determined the pharmacokinetic characteristics of YLT192 in male wistar rats. The pharmacokinetic parameters of YLT192 are summarized in Supplementary Table S2 and the plasma concentration versus time profile is shown in Supplementary Figure S3. After intravenous administration of 20 mg/kg of YLT192, YLT192 displayed a T_1/2_ of approximately 4.1 h, with a clearance of approximately 0.30 L/h/kg. Following oral administration of YLT192 at 50 mg/kg, the peak plasma concentration (C_max_) was approximately 4.1 μg/mL with a T_1/2_ of approximately 9.1 h. Overall, the oral bioavailability of YLT192 was 50.5%, which is relatively high, indicating that YLT192 had excellent pharmacokinetic parameters and can be orally administered once daily in *in vivo* studies.

### Anti-tumor activities of YLT192 *in vivo*

**Figure 5:**
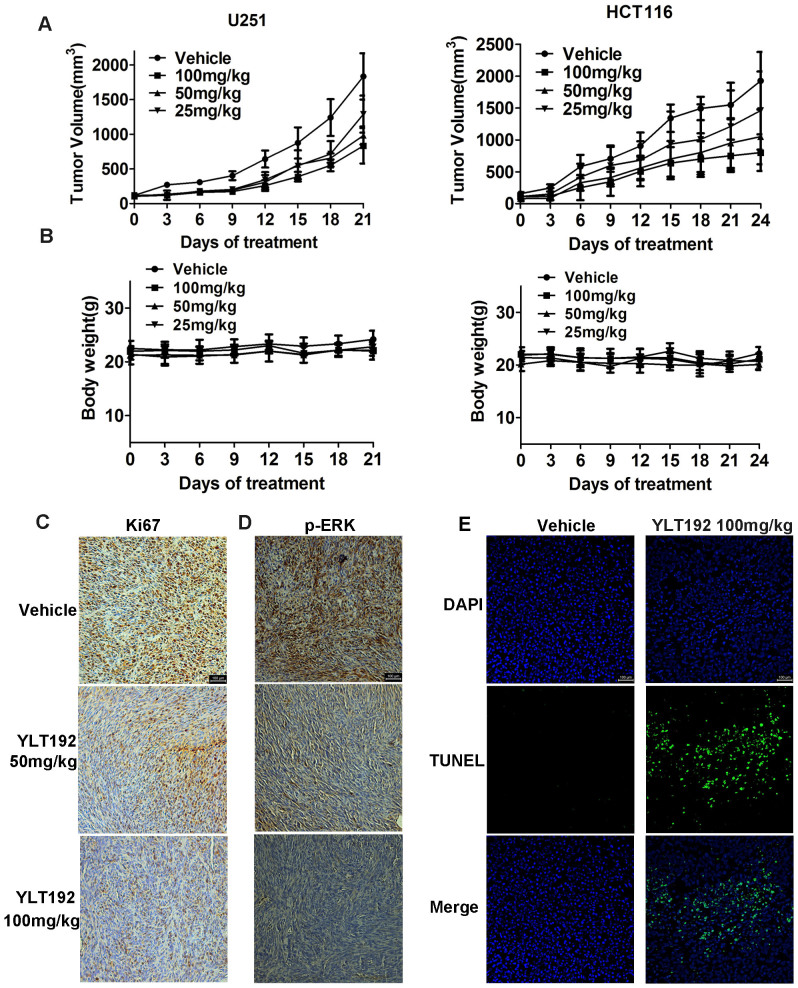
Anti-tumor activities of YLT192 *in vivo*. (A) U251 and HCT116 tumor-bearing mice were treated orally with YLT192 at 25, 50 and 100 mg/kg or vehicle daily, respectively. Treatment with YLT192 resulted in significantly tumor growth inhibition versus vehicle control. (B) Body weight changes in YLT192 and vehicle treated mice. There was no significant difference in body weight between YLT192 and vehicle treated group. n = 6 mice per group. For A and B, data are means ± SD. (C) and (D), YLT192 inhibited tumor cell proliferation in HCT116 tumor tissues *in vivo*. Mice bearing HCT116 tumor xenograft were treated with YLT192 at 50 and 100 mg/kg or vehicle and sacrificed at the end of the experiment. The tumor tissues were removed for further immunohistochemistry analysis using anti-Ki67 (C) and anti-p-p44/42 MAPK antibodies (D). (E) YLT192 induced tumor cell apoptosis in HCT116 tumor *in vivo*. Apoptosis was measured by TUNEL staining (n = 6 mice per group). Scale bars represent 100 μm.

As shown in Figure 5A, YLT192 significantly suppressed tumor growth, and tumor volumes of the 100 mg/kg YLT192-treated group were inhibited by 60% (U251) and 61% (HCT116) compared with the vehicle-treated group. Furthermore, YLT192 treatment was well tolerated, and there was no significant difference in weight between vehicle and YLT192 treated groups (Fig. 5B).

To better understand the mechanism of antitumor activities *in vivo*, immunohistochemistry was performed using tumor tissues isolated from HCT116 at the end of the treatment. We observed significant reduction in Ki67 staining in the YLT192 treatment group, indicating a decrease in proliferating cells in tumor tissues as shown in Figure 5C. At the same time, we observed a significant decrease in the phosphorylation of p44/42 MAPK (p-ERK) in the 100 mg/kg YLT192-treated group (Fig. 5D). Furthermore, we investigated the activity of YLT192 on apoptosis in HCT116 xenograft tumors by terminal deoxynucleotidyl transferase (dUTP) nick end labeling (TUNEL) staining. As shown in Figure 5E, 100 mg/kg of YLT192 increased apoptotic cells in the tumor tissues, which was consistent with the results of HCT116 cells *in vitro*. The data indicated that YLT192 could also induce apoptosis *in vivo*, which contributes to tumor growth suppression.

### Safety profile of YLT192

In the acute toxicity test, mortality, clinical signs, and body weight of the rats were measured for 14 days and no obvious changes were observed. Based on this result, the No Observed Adverse Effect Level (NOAEL) of YLT192 could be >3000 mg/kg. During the treatment of human xenografts in nude mice, we did not observed adverse effects, such as diarrhea or toxic death, in the YLT192-treated group. All the HCT116 model mice were sacrificed after 24 days of treatment. Hematological and serum biochemistry analysis and organ coefficients of the mice did not show any pathological changes (Supplementary Table 3–5). Moreover, microscopic examination of the heart, liver, spleen, lung, and kidney revealed no pathological changes after YLT192 treatment compared with the vehicle treatment group (Supplementary Fig. S4).

## Discussion

Tumor angiogenesis is pivotal for tumor growth and metastasis. Among the numerous factors involved in angiogenesis, VEGF and VEGFR2 are intensively studied and are proven to be promising targets against cancer. Targeting VEGFR2 with small molecule receptor tyrosine kinase inhibitors has demonstrated effectiveness in human cancer treatment^4^. Our group has been engaged in the design, screening, and synthesis of novel VEGFR2 inhibitors and one of them has been previously reported^24^. In the present study, we examined a small novel molecule YLT192 as a potent VEGFR2 inhibitor. The structure of YLT192 is different from other VEGFR2 inhibitors currently in clinical use and we obtained a patent for the inhibitor from the State Intellectual Property Office of the People's Republic of China. In the present study, we performed a broad preclinical characterization of the new VEGFR2 inhibitor, including biochemical, pharmacological, and toxicological profiles.

Kinase binding assays indicated that YLT192 selectively inhibited the kinase activity of VEGF2 and molecular docking verified these results. YLT192 had significant inhibitory effects on HUVEC function by suppressing HUVEC proliferation, migration, invasion, and tube formation *in vitro*. EC proliferation plays an important role in the process of angiogenesis from preexisting vessels, therefore, we examined whether YLT192 showed antiproliferative effects on ECs after stimulation by VEGF. The results showed that YLT192 markedly inhibited HUVEC proliferation stimulated by VEGF. The functions of vascular ECs rely on VEGFR2 signaling and VEGFR2 phosphorylation at Tyr1175 initiates downstream signaling pathways. Activation of the MAPK pathway has been shown to promote EC proliferation and motility^25^. Activation of AKT is necessary for angiogenesis. AKT regulates several EC functions such as migration and proliferation^26^, and stimulates the production of hypoxia-inducible factor (HIF)-α transcription factors; thus, promoting the secretion of VEGF^27^,^28^. STAT3 plays a critical role in angiogenesis and recent studies have identified it as a direct transcriptional activator of VEGF and HIF-α under hypoxic conditions, which initiate EC migration and angiogenesis^29^. In this study, YLT192 significantly inhibited VEGF-stimulated phosphorylation of VEGFR2 and downstream p44/42 MAPK, AKT, and STAT3 in HUVEC, indicating its ability to block angiogenesis (Fig. 2F). Moreover, cellular thermal shift assay validated that YLT192 bind to VEGFR2.

However, EC models lack the complex vessel systems of vertebrate animals^30^. To study the antiangiogenic effects of YLT192, we used zebrafish and athymic mouse models. Zebrafish have been used for antiangiogenic drug research and development because they can be a useful and cheap tool to investigate angiogenesis in a complete vertebrate system^31^,^32^. In the zebrafish assay, the fluorescent blood vessels of the transgenic EGFP-zebrafish facilitate image analysis. We observed that 5 μM YLT192 significantly inhibited angiogenic intersegmental blood vessel formation in zebrafish, which corresponds to capillary sprouting in mammalian animals. The alginate-encapsulated tumor cell assay is an exceptional model to study the mechanism of tumor angiogenesis inhibition and antiangiogenic drug development. The present study further confirmed that YLT192 could inhibit the angiogenic response induced by CT26 tumor cells in mice in a concentration-dependent manner.

Besides inhibiting tumor angiogenesis, YLT192 also had a direct inhibitory effect on tumor cells. It showed inhibitory effects in cancer cell viability assays and the IC_50_ values ranged from 6–30 μM. Moreover, YLT192 inhibited the proliferation of U251 and HCT116 cells, which are most sensitive to YLT192 treatment among the cancer cells treated, in a concentration-dependent manner as shown in the EdU-incorporation assay. Inducing apoptosis is a therapeutic approach to treat cancer and several anticancer drugs, including some VEGFR2 inhibitors can induce apoptosis^33^. YLT192 induced apoptosis in HCT116 cells. The increased forms of cleaved caspase-3 and expression of Bax after YLT192 treatment confirmed this conclusion. Activation of oncogenes and inactivation of tumor suppressor genes contributes to tumorigenesis. Hyperactivation of the RAS–RAF–MEK–MAPK (RAS–MAPK) pathway has been reported in human malignancies, making it a potential therapeutic target for cancer treatment^34^. MAPK is a well-known key downstream component of the RAS–MAPK pathway. Phosphorylated MAPK is translocated into the nucleus to transmit extracellular signals that regulate cell growth, differentiation, proliferation, apoptosis, and migration functions^34^,^35^. YLT192 significantly attenuated the phosphorylation of p44/42 MAPK in U251 and HCT116 cells, indicating its ability to block the oncogenic RAS–MAPK pathway. In addition, YLT192 inhibited other signaling pathways that promote the occurrence and development of cancer. The activity of STAT proteins, particularly STAT3 is constitutively elevated in a wide range of solid tumors and hematological malignancies, where it functions as a critical mediator of oncogenic signaling through transcriptional activation of genes related with apoptosis, cell cycle regulation, and angiogenesis^36^,^37^. Thus, inhibiting STAT3 has emerged as a promising molecular-targeted therapy strategy in cancer treatment^38^,^39^. The PI3K signaling pathway is one of the most frequently hyperactivated pathways in human cancer. AKT, a serine–threonine protein kinase, plays a critical role in the PI3K signaling axis, which is related to cell metabolism, proliferation, survival, and motility^40^. Therefore, inhibiting AKT has a potential role in the treatment of human cancer^41^. YLT192 greatly inhibited the activity of STAT3 and AKT in U251 and HCT116 cells. Moreover, YLT192 inhibited the phosphorylation of mTOR, a downstream regulator of AKT, which stimulates protein synthesis and cell growth leading to suppression of tumor growth^42^,^43^. Taken together, these results suggest that YLT192 is capable of inhibiting proliferation and inducing apoptosis in tumor cells; thus, contributing to its antitumor effects.

Not all small molecules that exhibit antitumor activities *in vitro* inhibit tumor growth *in vivo*. Therefore, we evaluated the effect of YLT192 on tumor growth in athymic mice. The good pharmacokinetic profile of YLT192 made it appropriate for once-daily oral administration. Therefore, we used this schedule in our preclinical *in vivo* work. YLT192 inhibited tumor growth in two human xenograft models after oral gavage. Histological studies of the tumor sections revealed that YLT192 also significantly reduced MVD and tumor cell proliferation. Moreover, similar to the apoptosis-inducing cancer cell effects observed *in vitro*, YLT192 also caused significant apoptosis in HCT116 tumor sections according to TUNEL assays.

In addition, YLT192 was well tolerated during the treatment of human cancer xenograft models. There were no adverse effects or clinical signs such as diarrhea, skin ulceration, and bleeding. In addition, body weight and organ coefficients of the mice were not affected. Moreover, microscopic examination showed that there were no pathological changes in the heart, liver, spleen, lung, and kidney after YLT192 treatment. In addition, hematological and serum biochemistry analysis of the mice revealed no pathological changes. Furthermore, there were no adverse effects observed during the acute toxicity test and the NOAEL of YLT192 could be >3000 mg/kg in rats. However, for some VEGFR2 inhibitors in clinical use, such as sorafenib and sunitinib, the maximal tolerance dose (MTD) is much lower than YLT192. The MTD of sorafenib and sunitinib is 500 mg/kg in rats, which is only 17% of the NOAEL value for YLT192 (http://www.accessdata.fda.gov/drugsatfda). Furthermore, there are toxicities in the gastrointestinal tract and liver after single doses of sorafenib and sunitinib. Although the effective dose for inhibition of tumor growth was slightly higher, the excellent safety profile of YLT192 indicated it has potential as a novel anticancer agent with limited or no off-target toxicity.

Taken together, our study indicated that YLT192, a novel, well-tolerated, and orally active VEGFR2 inhibitor, effectively inhibited tumor angiogenesis and growth *in vivo*. YLT192 and its derivatives will make promising drug candidates for cancer therapy. Further investigation of YLT192 is underway for its structural optimization to provide a promising drug candidate for cancer therapy.

## Methods

### Materials

3-(4, 5-dimethylthiazol-2-yl)-2,5-diphenyltetrazolium (MTT), dimethyl sulfoxide (DMSO) were purchased from Sigma Chemical Co. (St. Louis, MO). Cell-Light™ EdU DNA Cell Proliferation Kit was purchased from RiboBio Co., Ltd. (Guangzhou, China). Recombinant human VEGF_165_ was obtained from PeproTech Inc. Recombinant human EGF was purchased from ProSpec Company. Human basic fibroblast growth factor (bFGF), anti-CD31 antibody, and Matrigel were purchased from BD Bioscience (San Diego, CA). The primary antibodies were acquired from Cell Signaling Technology (Beverly, MA). Annexin V-FITC apoptosis detection kit was purchased from Roche (Indianapolis, IN). All of the chemicals used in this study were of analytical and culture grade. For all *in vitro* assays and zebrafish studies, YLT192 was dissolved in DMSO as a 40 mM stock solution and diluted in the relevant assay media. For *in vivo* experiments, YLT192 was dissolved in 25% (v/v) aqueous Cremophor EL/ethanol (50:50; Sigma Cremophor EL, 100% ethyl alcohol) and dosed at 0.1 mL/10 g of body weight. For all *in vitro* assays, medium with 0.1% DMSO served as vehicle control.

### Cell lines and cell culture

The human hepatocellular cell lines Bel-7402 and SMMC-7721 were obtained from China Center for Type Culture Collection (CCTCC, Wuhan, China). Human umbilical vein endothelial cells (HUVECs) were isolated from human umbilical cord, which was supported by Department of Gynecology and Obstetrics, West China Second Hospital, Sichuan University, Chengdu, Sichuan, China. All the other cell lines were obtained from the American Type Culture Collection (Manassas, VA, USA). All the cells except HUVEC were cultured in RPMI1640 or DMEM supplemented with 10% FBS (Gibco, Grand, NY). HUVECs were grown in endothelial basal medium-2 (EBM-2) supplemented with SingleQuots Kit containing VEGF and other growth factors (LONZA). HUVECs at passages 3–8 were used in all the studies.

### Molecular modeling

The X-ray crystal structure of VEGFR2 complexed with sorafenib (PDB ID: 4ASD) was used as the reference receptor for the docking study. The docking program GOLD (version 5.0) was adopted. The preparation of VEGFR2 structure, including adding hydrogen atoms, removing water molecules, and assigning Charmm force field, were carried out by using Discovery Studio 3.1 software package. A sphere containing the residues in VEGFR2 that stay within 11 Å from sorafenib were defined as the binding site. GoldScore was selected as the score function, and the other parameters were set as default. A total of 20 docking poses were retained. Finally, the root-mean's quare deviation (RMSD) between docking poses were calculated.

### EdU incorporation assay

EdU is a thymidine analogue used to label proliferating cells which can incorporate into replication DNA when cells are dividing^44^. Cells growing in 96-well plates were treated with different concentrations of YLT192 for 24 hours, and then assayed with Cell-Light™ EdU DNA Cell Proliferation Kit according to the manufacture's instructions. Each assay was replicated 3 times.

### Wound healing migration and transwell invasion assay

HUVECs were allowed to grow into full confluence in 24-well plates and then wounded by scratching with pipette tips and washed with PBS. Fresh EGM2 with growth factors was added with different concentrations of YLT192 or vehicle. Images were taken by an OLYMPUS inverted digital camera after 24 hours incubation. Transwell assay was conducted as described previously with some modifications^45^. Matrigel diluted 1:3 in serum-free medium was added to the top chamber of 24-well transwell plate (Millipore). After Matrigel polymerization, the bottom chambers were filled with 600 μL EGM2 medium containing various growth factors. The top chambers were seeded with 100 μL EBM2 medium (without growth factors) and HUVECs (4 × 10^4^ cells per well). Immediately, 100 μL EBM2 medium with various concentrations of YLT192 was added to the upper chamber. After 24 hours, invasion was stopped by scraping nonmigrated cells on the top chamber with a cotton swab. Migrated cells were fixed in 4% paraformaldehyde and stained with 0.05% Crystal Violet. Images were taken using a ZEISS digital microscope and invading cells were counted by manual counting. Vandetanib served as positive control. The assays were replicated 3 times.

### Capillary-like tube formation assay

The anti-angiogenesis ability of YLT192 *in vitro* was evaluated using capillary tube formation assay as described previously^46^. Briefly, 96-well plate was pre-incubated with Matrigel at 37°C for 45 minutes. Then HUVECs (2 × 10^4^ cells) suspended in EGM2 medium were seeded onto the Matrigel. They were treated with various concentrations of YLT192. After 6 hours incubation at 37°C, tube formation was assessed with an OLYMPUS inverted digital camera in low power fields (50×). Vandetanib served as positive control. The assay was replicated 3 times.

### Target engagement assay

The ability of YLT192 to interact with, and thereby stabilize the target in intact HUVECs, was evaluated as described by Molina et al.^22^ with some modifications. Briefly, subconfluent HUVECs were incubated with 40 μM YLT192 or vehicle for 3 h. Then the cells were detached with trypsin and collected in TBS containing complete protease inhibitor cocktail. The cells were divided into 4 aliquots and heated individually at 40, 43, 46, 49°C for 3 minutes. Subsequently, the cell suspensions were freeze-thawed three times using liquid nitrogen. Soluble proteins were separated from the precipitated fraction by centrifugation at 17,000 g for 20 min. The proteins were then detected with immunoblot analysis using the VEGFR2-antibody.

### Alginate-encapsulated tumor cell assay

To determine whether YLT192 could inhibit angiogenesis induced by tumor cells *in vivo*, we did the alginate-encapsulated tumor cell assay as described previously^47^. Briefly, alginate beads containing 5 × 10^4^ CT26 cells each were formed and implanted subcutaneously into both the dorsal flank of athymic mice. Then the mice were treated with 100 and 50 mg/kg YLT192 or vehicle every day from the second day. Two weeks later, fluorescein isothiocyanate (FITC)-dextran solution (Sigma, St. Louis, MO) was injected into the later tail vein of the mice at the dosage of 100 mg/kg. Twenty minutes after the injection, the alginate beads were removed and photographed. The content of FITC-dextran in the beads was quantified as described previously.

### Drug studies in zebrafish

The transgenic zebrafish (FLK-1: EGFP) was used in the experiments. The embryos were grown and maintained as described previously^48^. Fifteen hours post fertilization (hpf), zebrafish embryos were treated with indicated concentrations of YLT192 or vehicle. At 30 hpf, zebrafish was anesthetized with 0.01% tricaine and fluorescent images of the embryos were captured using the Fluorescent Microscope (Carl Zeiss Micro Imaging Inc.). The assay was replicated 3 times.

### Subcutaneous xenograft models

All animal experiments were approved and conducted by the Institutional Animal Care and Treatment Committee of Sichuan University in China (Permit Number: 20121205). HCT116 and U251 tumors were established by injecting 1 × 10^7^ cells (100 μL) into the dorsal area of 7–8 week old female athymic nude mice (balb/c, nu/nu). When tumors reached a volume of about 100 mm^3^, mice were randomized (6 per group) and YLT192 (25–100 mg/kg) or vehicle were given once daily by oral gavage. Tumor growth and body weight was measured every three days during the treatment. Tumor volumes were calculated using the formula as follow: volume (mm^3^) = 0.5 × length (mm) × width (mm)^2^. Growth inhibition was calculated from the start of treatment by comparison of the mean change in tumor volume for vehicle and treated group as before^14^.

### Histopathology and immunohistochemistry

Tumour tissues obtained from the mice bearing HCT116 tumors were subjected to immunohistological analysis. Briefly, at the end of the treatment, mice were killed and tumor tissues were obtained. Then CD31 staining was conducted using frozen sections of tissue embedded in optimum cutting temperature^49^ and immunohistological detection of anti-Ki67 and p-p44/42 MAPK were performed according to the manufacture's instructions using paraffin-embedded sections of tumor^50^. Apoptotic cells in the tumor tissue were detected by terminal deoxynucleotidyl transferase-mediated dUTP nick end-labeling (TUNEL) staining using an apoptotic cell detection kit (Merck Millipore).

### Toxicity evaluation

Acute toxicity test was done to investigate the safety profiles of YLT192 on rats. Briefly, 7–8 week old female and male rats (n = 10, respectively) were orally administrated with YLT192 at 3000 mg/kg. Then clinical symptoms of the rats including mortality, body weight and gross findings were observed once daily for 14 days. On day 14, the rats were sacrificed and examined by necropsy.

During the treatment of human colorectal carcinoma HCT116 xenograft on nude mice, they were also observed every day to investigate the side effects and toxicities after YLT192's longer time administration. Finally, serum biochemistry, hematological analysis, organ coefficients and histological examinations of heart, liver, spleen, lung, and kidney were carried out after dissection. Serum chemistry and hematological analysis was carried out.

### Statistical analysis

Data were expressed as mean ± SD/SEM. Statistical analysis was conducted using SPSS 13.0 software. Data were analyzed by using one-way analysis of variance (ANOVA). Two-tailed tests were used for all hypothesis tests in the present study. Differences were considered statistically significant when P < 0.05.

### Ethics statement

The methods were carried out in accordance with the approved guidelines. The isolation procedure of HUVECs was approved by the institutional review board of the Medical Faculty at the West China Second Hospital, Sichuan University.

## Author Contributions

Y.X., Y.Q.W., Y.L.Z. and L.T.Y. conceived and designed the experiments. Y.X., X.J.S., G.B.L., S.Y.D. and S.Z. performed most of the biological experiments. Y.X., D.L.L., T.H.Y., Y.Z.X. and H.J.L. analyzed the data. N.N.M., L.L. and Y.X.Z. conducted the synthesis of YLT192. J.Z., Q.L. and Y.L.P. contributed some of the materials. Y.X., X.J.S. and T.H.Y. wrote the main manuscript text. All the authors reviewed the manuscript.

## Supplementary Material

Supplementary Information Supplementary information

## Figures and Tables

**Table 1:** *In vitro* profile of YLT192 against a panel of 29 kinases. The assays were performed in two independent experiments. Data are means ± SD

Kinase	inhibition rate at 1 μM (%)
VEGFR2	93 ± 3
Flt3	−6 ± 2
PDGFRα	5 ± 3
PDGFRβ	7 ± 8
cKit	6 ± 3
FGFR1	21 ± 3
FGFR2	19 ± 4
FGFR3	4 ± 6
Plk1	1 ± 3
Aurora-A	−1 ± 4
Aurora-B	23 ± 5
Ret	23 ± 3
Axl	−7 ± 7
Met	−2 ± 6
PKA	−10 ± 4
Fms	35 ± 11
CDK2/cyclinE	−9 ± 3
IR	3 ± 3
CDK6/cyclinD3	0 ± 4
EGFR	1 ± 6
PI3K	0 ± 2
IKKβ	2 ± 6
JNK3	3 ± 5
mTOR	0 ± 6
ALK	−4 ± 3
LCK	3 ± 6
GSK3β	4 ± 6
JAK3	−7 ± 4
c-RAF	3 ± 3
