# Supplementary material for: YLT192, a Novel, Orally Active Bioavailable Inhibitor of VEGFR2 Signaling with Potent Antiangiogenic Activity and Antitumor Efficacy in Preclinical Models
Source: Sci Rep. 2014 Aug 12;4:6031. doi: 10.1038/srep06031 (PMC4129416; doi:10.1038/srep06031)
Supplement: Supplementary Information [file srep06031-s1.pdf]

Supplementary information

YLT192, a Novel, Orally Active Bioavailable Inhibitor of VEGFR2 Signaling with Potent Antiangiogenic Activity and Antitumor Efficacy in Preclinical Models

Yong Xia[1†], Xuejiao Song[1†], Deliang Li[1], Tinghong Ye[1], Youzhi Xu[1], Hongjun Lin[1], Nana Meng[1], Guobo Li[1], Senyi Deng[1], Shuang Zhang[1], Li Liu[1], Yongxia Zhu[1], Jun Zeng[1], Qian Lei[1], Youli Pan[1], Yuquan Wei[1], Yinglan Zhao[1]*, Luoting Yu[1]*

[1]State Key Laboratory of Biotherapy and Cancer Center, West China Hospital, West China Medical School, Sichuan University, Chengdu, 610041, China;

†These authors contributed equally to this work.

Supplementary Table S1-S5

Supplementary Figures S1-S8

Supplementary Table S1: IC50 values (μM) for inhibition of cell growth by 48 hour treatment with YLT192. Data are means ± SD, n=3.

cell line	cell type	IC_{50}(μM)
U251	human glioblastoma	6.5 ±0.8
HCT116	human colorectal carcinoma	7.4 ±0.5
A431	human pidermoid carcinoma	7.7 ±0.9
BxPC-3	human pancreatic cancer	8.2 ±0.6
Hep G2	human hepatocellular carcinoma	7.9 ±0.5
SW620	human colorectal carcinoma	7.9 ±1.7
SKOV-3	human ovarian carcinoma	8.2 ±1.5
LS147T	human colorectal carcinoma	9.0 ±1.1
PANC-1	human pancreatic cancer	9.8 ±1.9
A2058	human melanoma	10.7 ±1.1
BEL7402	human hepatocellular carcinoma	11.3 ±1.1
MDA-MB-231	human mammary carcinoma	11.9 ±2.1
SMMC-7721	human hepatocellular carcinoma	12.1 ±1.6
A375	human melanoma	12.4 ±1.1
A875	human melanoma	14.1 ±2.1
SPC-A1	human lung cancer	16.4 ±1.4
MCF-7	human mammary carcinoma	16.8 ±1.7
Hela	human epithelial carcinoma	18.9 ±2.4
B16	mouse melanoma	21.2 ±2.1
MV4-11	human acute myeloid leukemia	21.9 ±1.9
DU145	human prostate cancer	22.8 ±2.6
COLO-205	human colorectal carcinoma	23.0 ±1.5
NIH-H1975	human lung cancer	27.3 ±1.2

Supplementary Table S2: Pharmacokinetic parameters for YLT192 in rats (n=6 mice per group). Data are means ± SD.

Route	Dose (mg/kg)	C_{max} (μg/mL)	AUC(0-∞) (mg/L*h)	$T_{1/2}$ （h）	CL (L/h/kg)	Vz (L/kg)	F (%)
I.V.	20	—	65.9±6.2	4.1±2.1	0.06±0.01	1.79±0.24	—
P.O.	50	4.1±0.4	83.3±21.9	9.1±4.1	0.06±0.02	7.84±1.12	50.5%

Note: I.V.=intravenous, P.O.=orally administration, C_{max} =maximum plasma concentration, AUC=area under the curve, $T_{1/2}$=half life of the inhibitor, CL=clearance , Vz=volume of distribution, F=bioavailability

Supplementary Table S3: The hematological parameters of mice after the treatment of HCT116 xenograft in nude mice (n=6 mice per group). Data are means ± SD.

	Vehicle	YLT192 100mg/kg	P value
WBC (109/L)	9.87±2.82	8.15±3.63	0.38
RBC (1012/L)	8.76±1.15	8.61±0.56	0.78
HGB (g/L)	118.00±14.16	112.83±8.42	0.46
HCT	0.50±0.07	0.48±0.04	0.55
PLT(109/L)	306.83±64.26	364.17±127.00	0.35
PCT(%)	0.27±0.05	0.32±0.11	0.42
MCV(fL)	56.95±3.10	55.63±2.94	0.47
MCH(pg)	13.47±0.42	13.10±0.62	0.26
MCHC (g/L)	237.33±13.28	235.50±8.69	0.78
RDW(%)	15.02±0.57	15.25±0.52	0.48
MPV(fL)	9.13±0.38	8.82±0.44	0.22
PDW(%)	13.87±0.30	13.7±0.57	0.54
LY(%)	25.87±5.23	27.53±11.52	0.75
MO(%)	10.32±4.59	10.17±4.58	0.96
GR(%)	63.82±6.77	62.30±14.77	0.82
LY(109/L)	2.60±1.33	2.37±1.49	0.77
MO(109/L)	0.98±0.52	0.87±0.61	0.73
GR(109/L)	6.28±1.76	4.92±2.41	0.29

Supplementary Table S4: The serum biochemistry parameters of mice after the treatment of HCT116 xenograft in nude mice (n=6 mice per group). Data are means ± SD.

	Vehicle	YLT192 100mg/kg	P value
ALB (g/L)	26.4±10.0	31.6±1.6	0.21
ALP (U/L)	161.0±9.8	151.2±6.4	0.06
ALT (U/L)	37.3±4.5	45.5±6.8	0.06
AST(U/L)	199.3±72.1	193.4±38.2	0.83
CK (U/L)	836.6±350.6	1152.1±473.4	0.17
CREA (uM)	11.6±3.5	13.0±3.0	0.31
TBIL (uM)	1.38±0.49	0.64±0.39	0.02
GLU (mM)	7.8±2.9	6.6±1.4	0.22
TP (g/L)	48.8±1.8	50.5±3.4	0.22
TG (mM)	1.22±0.48	0.95±0.22	0.16

Supplementary Table S5: The organ coefficients of mice after the treatment of HCT116 xenograft in nude mice (n=6 mice per group). Data are means ± SD.

	Vehicle	YLT192 100 mg/kg	P value
Heart %	0.51±0.09	0.55±0.06	0.30
Liver %	5.93±0.69	6.45±0.16	0.10
Spleen %	0.83±0.18	0.71±0.14	0.24
Lung %	0.93±0.55	1.16±0.76	0.55
Kidney %	1.46±0.08	1.46±0.12	0.92

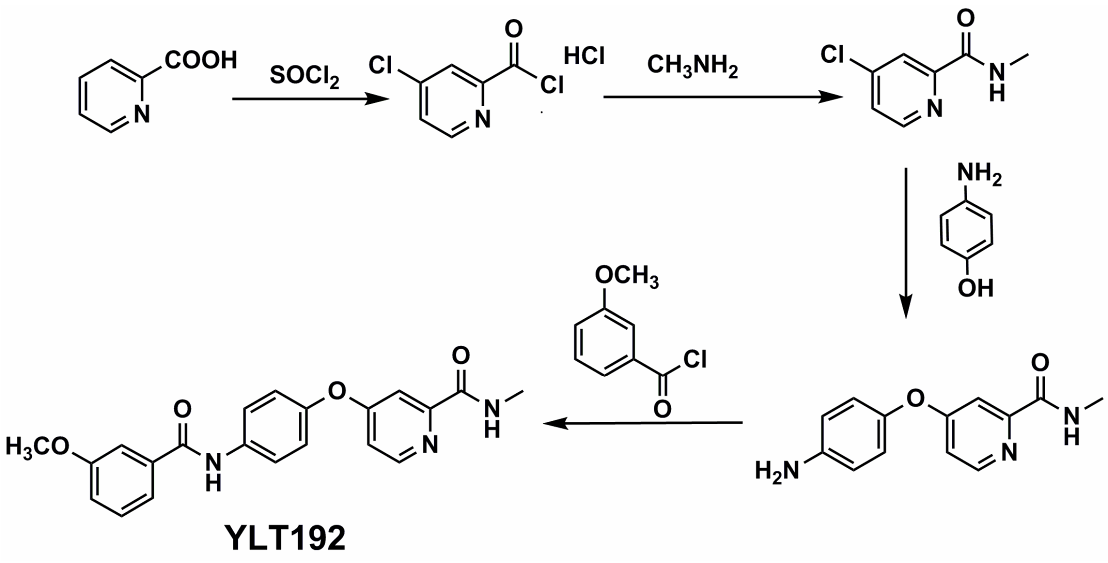

Supplementary Figure S1. A procedure for the synthesis of YLT192. YLT192 was synthesized as following step. Briefly, thionyl chloride was added to a mixture of sodium bromide, 2-picolinic acid and chlorobenzene. Then the addition of methylamine yielded the intermediate (4-chloro-N-methylpicolinamide) as a white solid. Potassium tert-butoxide and 4-aminophenol were dissolved in N,N-dimethylformamide. The reaction was stirred at room temperature for 2h and 4-chloro-N-methylpicolinamide and potassium carbonate were added, and then stirred at 85 °C under nitrogen for 15 h. The reaction mixture was then diluted with water and extracted with ethyl acetate. The organic extracts were dried over anhydrous $MgSO_4$ and concentrated under reduced pressure. The residue and anhydrous potassium carbonate were suspended in THF, and then 4-methoxybenzoyl chloride was added drop-wise at 0–5 °C. Ethyl acetate and brine were added to the reaction mixture, and the organic layer was separated and concentrated in vacuo. The residue was purified by column chromatography to afford YLT192.

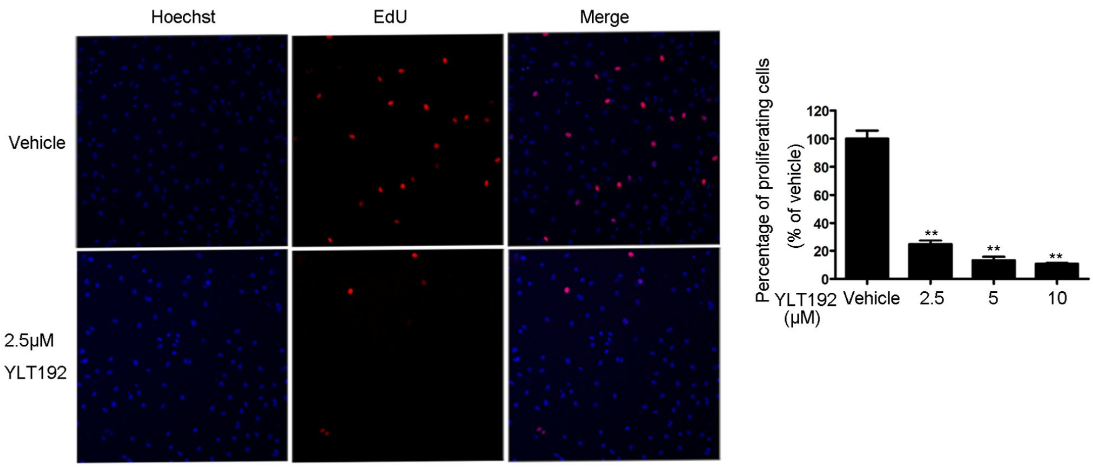

Supplementary Figure S2. YLT192 inhibited VEGF-stimulated HUVECs proliferation in the EdU incorporation assay. The EdU-positive(red fluorescent staining) cells and Hoechst staining(blue fluorescent staining) cells represented proliferating and total cells, respectively(100×). Quantification was shown on the right panels. Data represent the mean ± SEM of three different experiments. Each experiment was performed in duplicate. *P<0.05, **P<0.01.

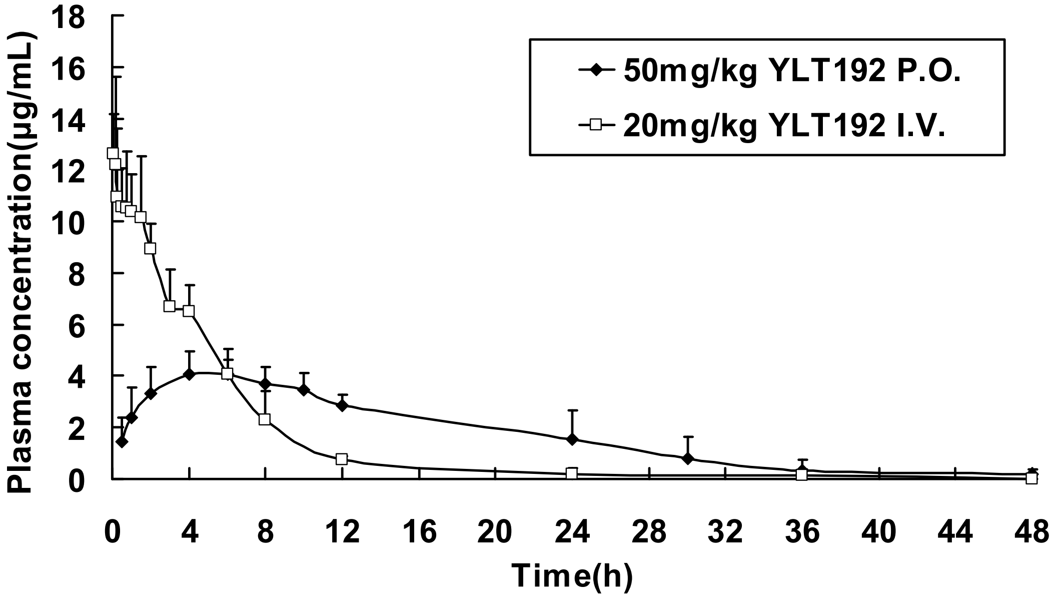

Supplementary Figure S3. Mean plasma concentration-time curves of YLT192. After I.V.(20mg/kg) and P.O.(50mg/kg) administration of YLT192 in wistar male rats, the concentration of YLT192 in the plasma was determined by HPLC (n=6 mice per group). Data are means ± SD.

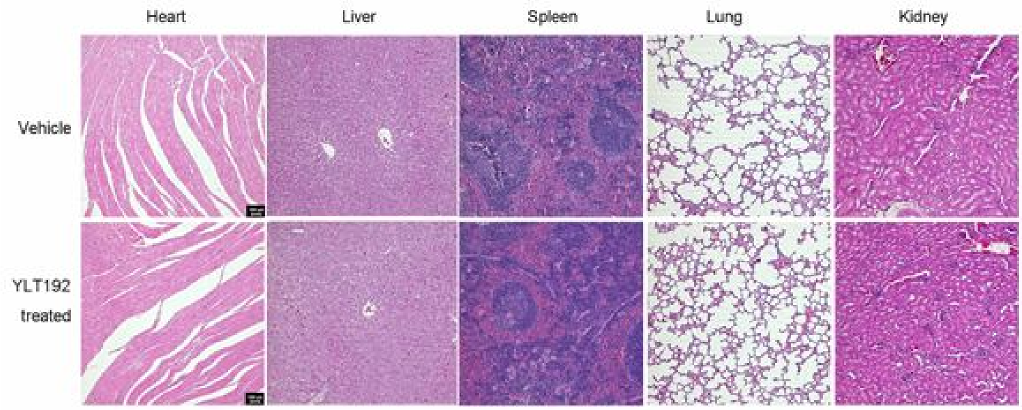

Supplementary Figure S4. YLT192 did not cause obvious pathologic abnormalities in normal tissues. After the treatment of HCT116 xenograft, heart, liver, spleen, lung and kidney of the mice were isolated and stained with H&E (n=6 mice per group). Scale bars represent 50 μm.

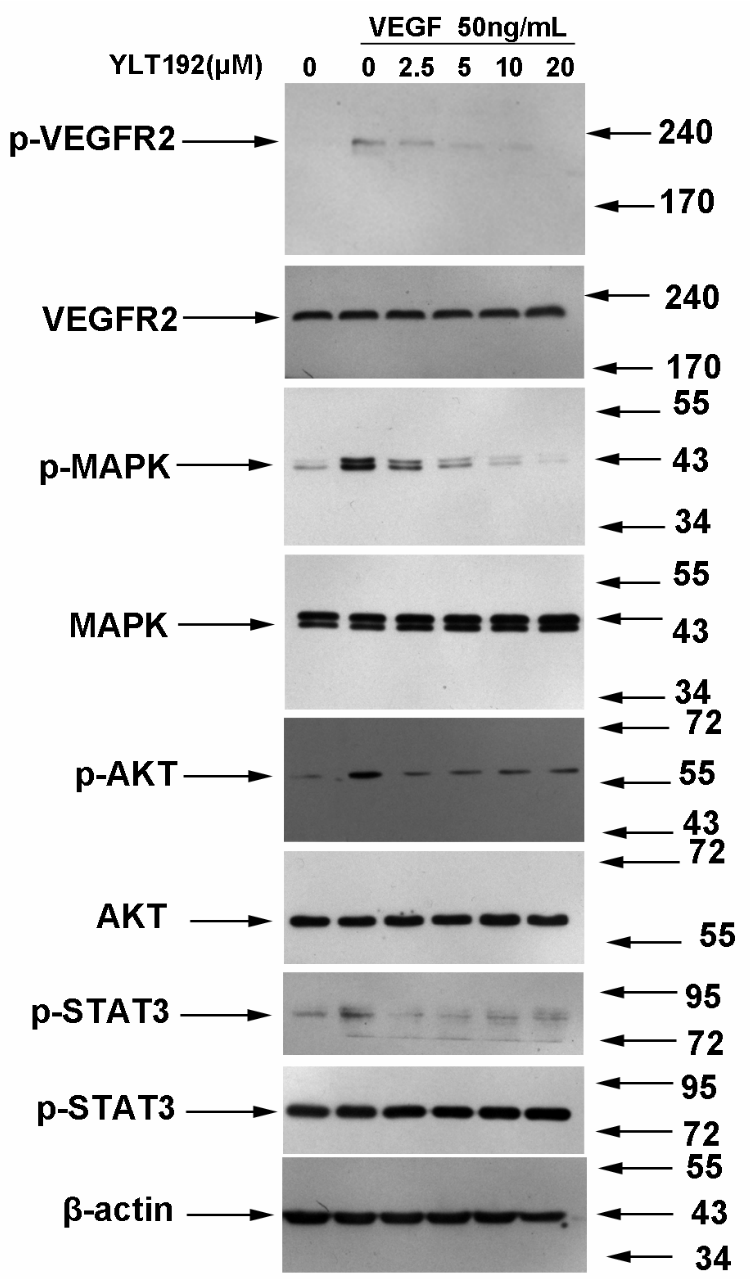

Supplementary Figure S5. **Inhibitory Effects of YLT192 on VEGFR2 signaling in HUVECs.** YLT192 inhibited VEGF-induced phosphorylation of VEGFR2 and its downstream signal regulator p44/42 MAPK, STAT3 and AKT in HUVECs. One representative Western blot of three is presented. Each has the expression of β-actin as internal control. These cropped blots are used in the main figure (Figure 2) and these full-length blots are included in the supplementary figure.

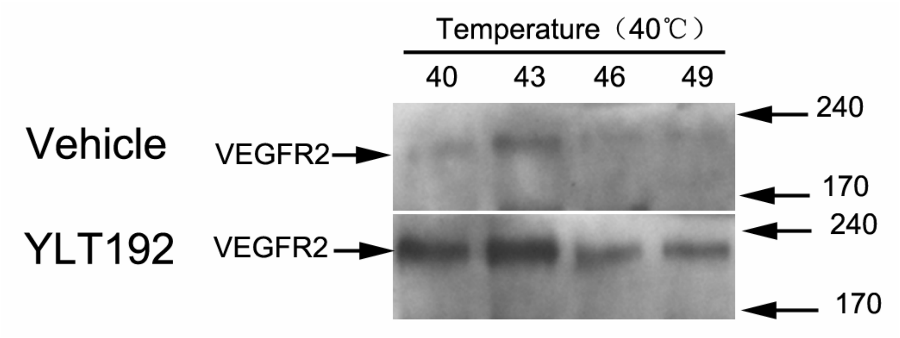

Supplementary Figure S6. Cellular thermal shift assay showing VEGFR2 target engagement by YLT192 in intact HUVECs. One representative Western blot of two is presented. These cropped blots are used in the main figure (Figure 2) and these full-length blots are included in the supplementary figure.

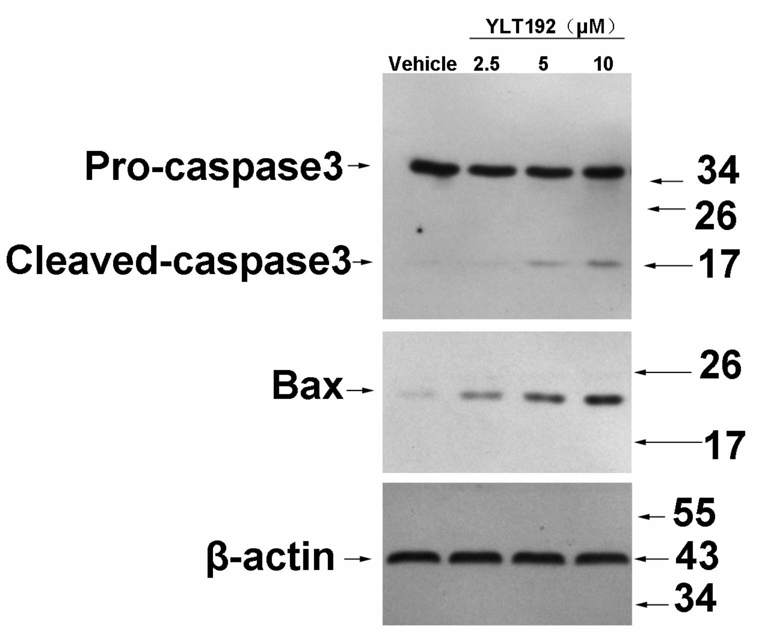

Supplementary Figure S7. **YLT192 induced apoptosis of HCT116 cells.** HCT116 cells were treated with YLT192 or vehicle for 48 hours and then typical apoptosis-related proteins caspase 3 and Bax were detected by western blot. Each has the expression of β-actin as internal control. These cropped blots are used in the main figure (Figure 4) and these full-length blots are included in the supplementary figure.

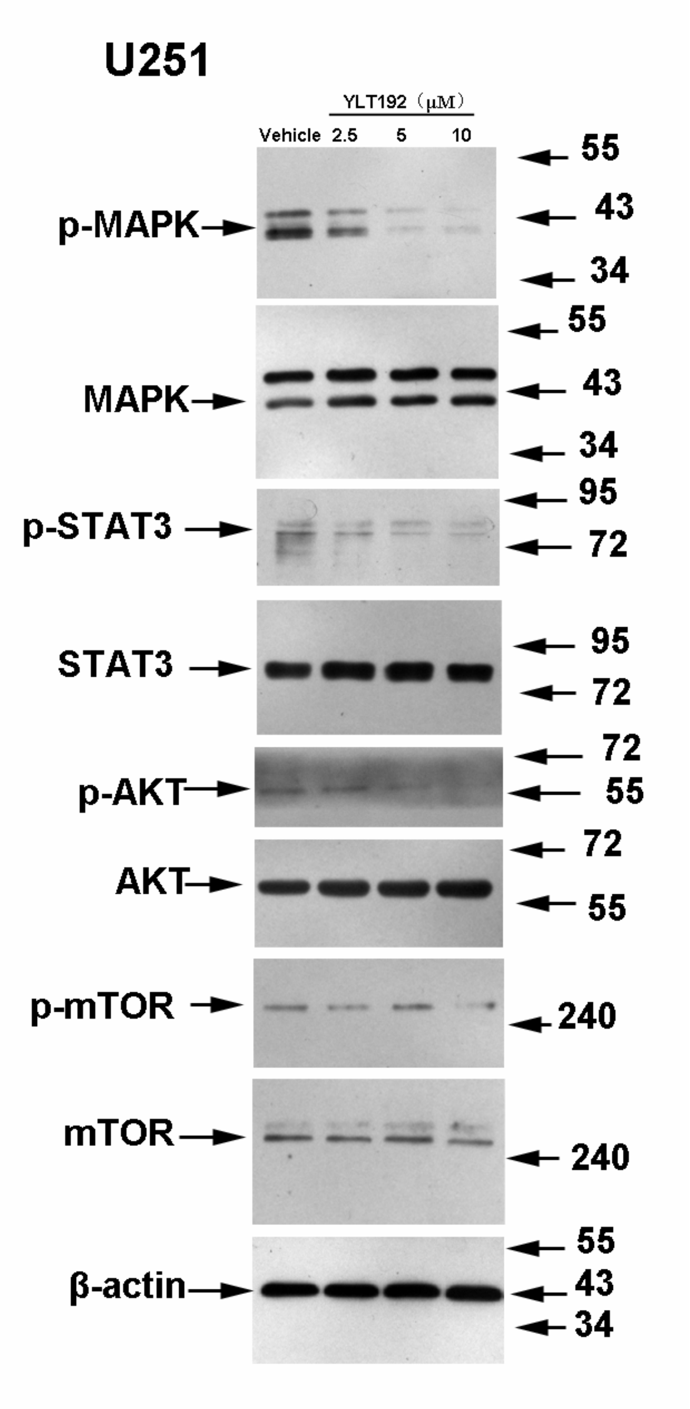

U251
YLT192（μM）
Vehicle 2.5 5 10
p-MAPK
MAPK
p-STAT3
STAT3
p-AKT
AKT
p-mTOR
mTOR
β-actin
55
43
34
55
43
34
95
72
95
72
72
55
72
55
240
240
55
43
34

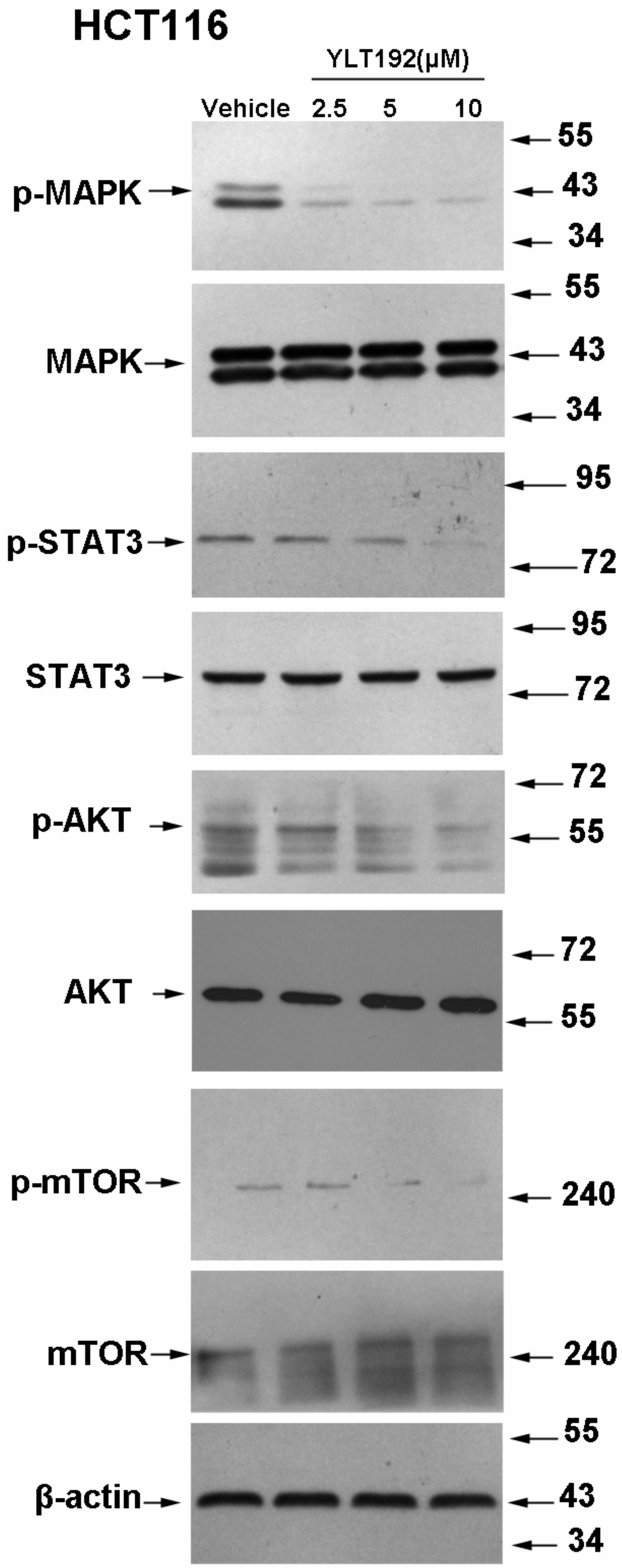
HCT116
YLT192(μM)
Vehicle
2.5
5
10
p-MAPK
MAPK
p-STAT3
STAT3
p-AKT
AKT
p-mTOR
mTOR
β-actin
55
43
34
95
72
240

Supplementary Figure S8. **YLT192 inhibited oncogenic signaling pathways in tumor cells *in vitro***. U251 and HCT116 cells were treated with YLT192 or vehicle for 48 hours and the expression of protein in different signaling pathways were detected by western blot with specific antibodies. Blots are representative of three experiments. Each has the expression of β-actin as internal control. These cropped blots are used in the main figure (Figure 4) and these full-length blots are included in the supplementary figure.
